# Sex differences in self‐reported attention‐deficit/hyperactivity disorder symptoms in clinical and population‐based cohorts

**DOI:** 10.1002/jcv2.70012

**Published:** 2025-03-26

**Authors:** Berit S. Solberg, Maj‐Britt Posserud, André Høberg, Johanne T. Instanes, Liv G. Kvalvik, Amalie Austgulen, Ammal Khan, Astri J. Lundervold, Anne Halmøy, Rolf Gjestad, Kari Klungsøyr, Jan Haavik

**Affiliations:** ^1^ Department of Biomedicine University of Bergen Bergen Norway; ^2^ Child‐ and Adolescent Psychiatric Outpatient Unit Hospital Betanien Bergen Norway; ^3^ Division of Psychiatry Haukeland University Hospital Bergen Norway; ^4^ Department of Clinical Medicine University of Bergen Bergen Norway; ^5^ Gillberg Neuropsychiatry Centre Institute of Neuroscience and Physiology Sahlgrenska Academy University of Gothenburg Gothenburg Sweden; ^6^ Department of Global Public Health and Primary Care University of Bergen Bergen Norway; ^7^ Department of Biological and Medical Psychology University of Bergen Bergen Norway; ^8^ Kronstad District Psychiatric Center Division of Psychiatry Haukeland University Hospital Bergen Norway; ^9^ Research Department/Centre for Research and Education in Forensic Psychiatry Division of Psychiatry Haukeland University Hospital Bergen Norway; ^10^ Center for Crisis Psychology Faculty of Psychology University of Bergen Bergen Norway; ^11^ Division of Mental and Physical Health Norwegian Institute of Public Health Bergen Norway; ^12^ Bergen Center for Brain Plasticity Division of Psychiatry Haukeland University Hospital Bergen Norway

**Keywords:** ADHD diagnosis, ADHD symptoms, adult ADHD, sex differences

## Abstract

**Background:**

Although the childhood prevalence of ADHD is higher in males than in females, some studies suggest that females report more severe impairment related to ADHD symptoms than males. The reason for this gender paradox is poorly understood. Here we explored sex differences in self‐reported ADHD symptoms among adolescents and adults with and without an ADHD diagnosis across clinical and population‐based Norwegian cohorts.

**Methods:**

We analysed population‐based data from the Norwegian Mother, Father, and Child Cohort Study (MoBa) (*N* = 90,816; 62% females, mean age 33.4 years), the youth@hordaland study (y@h) (*N* = 10,257; 53% females, mean age 17.4 years), and the ADHD in Norwegian Adults (ANA) project (*N* = 1677; 55% females, mean age 29.0 years). The Adult ADHD Self‐Report Scale Screener (ASRS‐6) was used as an outcome measure to define symptom severity of ADHD. Linear regression analyses were used to explore sex differences in ASRS‐6 scores between individuals with and without a self‐reported ADHD diagnosis (∆). Age‐adjusted, unstandardized regression coefficients (b) with 95% confidence intervals (CI) were reported.

**Results:**

Participants with ADHD reported higher ASRS‐6 scores than those without ADHD across all three cohorts. The ASRS‐6 scores were highest in the youngest participants, particularly in females from the y@h cohort. The difference in ASRS‐6 scores between those with and without an ADHD diagnosis was larger among females than males within all cohorts, at a statistically significant level both in y@h and ANA (MoBa: ∆females: *b* = 4.6 (95%CI 3.8; 5.4); ∆males: *b* = 4.2 (3.5; 5.0); y@h: ∆females: *b* = 5.1 (4.0; 6.2); ∆males: *b* = 3.2 (2.1; 4.2); ANA: ∆females: *b* = 8.6 (8.1; 9.2); ∆males: *b* = 6.5 (5.9; 7.2).

**Conclusions:**

In both population‐based and clinical cohorts, representing different age groups and study designs, we found a larger mean difference in reported ADHD symptoms between adolescents and adults with and without ADHD among females than males, suggesting barriers for females related to referral, assessment, and/or diagnosis of ADHD.

## Graphical abstract

There is an apparent gender paradox in ADHD; the childhood prevalence is higher in males than females but adult females with ADHD are reporting more severe impairment. We found that the relative difference in symptom levels between individuals with and without ADHD was larger in females than males, both in a clinical sample and in the general population of ∼102,000 adolescents and adults. The relatively higher level of ADHD symptoms in females could suggest sex related barriers for obtaining a clinical diagnosis of ADHD.

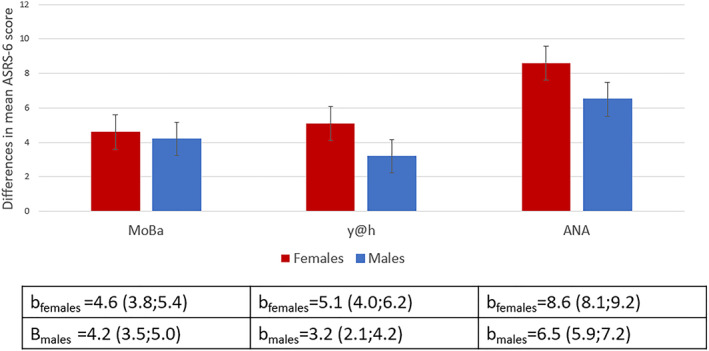

**Key Points**

- *What’s known* – There is an apparent gender paradox in ADHD; the childhood prevalence is higher in males than in females but adult females with ADHD are reporting more severe impairment.
- *What’s new* – The relative difference in symptom levels between individuals with and without ADHD was larger in females than males, both in a clinical cohort and in the general population
- *What’s relevant* – The relatively higher level of ADHD symptoms in females could suggest barriers for females related to referral, assessment, and/or diagnosis of ADHD.

A diagnosis of attention‐deficit/hyperactivity disorder (ADHD) (American Psychiatric Association, 2013) is a categorical construct; however, ADHD symptoms are continuously distributed in the general population, with the clinical ADHD diagnosis typically representing the extreme end of that distribution (Demontis et al., 2019; Greven et al., 2016; Rössler, 2013). Sex differences related to the ADHD diagnosis have been reported, with a higher prevalence of ADHD diagnoses in males than in females in children (male/female ratio of 9 – 4:1) (Biederman et al., 2004; Bitter et al., 2010; Robison et al., 2008), with a more even sex balance towards adulthood (American Psychiatric Association, 2013; Kessler et al., 2006). However, in studies from the general population, males typically report higher ADHD symptom scores than females, while symptom severity in clinically diagnosed males is similar to the severity level in females with ADHD (Arnett et al., 2015; F. D. Mowlem et al., 2019; Mowlem et al., 2019). In comparison, females without ADHD tend to report less ADHD symptoms than males without ADHD (Vildalen et al., 2019).

Other studies have shown that females with ADHD report more severe impairment at similar ADHD symptom levels than males with the diagnosis (Biederman et al., 2004; Fedele et al., 2012; Hinshaw et al., 2012; Vildalen et al., 2019), have more comorbid internalizing disorders like anxiety and depression (Rucklidge, 2010; Solberg et al., 2018; Wilens et al., 2009; Williamson & Johnston, 2015), and more often have a history of treatment for other psychiatric disorders (Halmoy et al., 2009; Skoglund et al., 2023). Further, females with ADHD typically report a greater impact of negative life events compared to males with ADHD (Attoe & Climie, 2023; Garcia et al., 2012; Young et al., 2020).

These differences have been suggested to indicate that females, at least in childhood, experience barriers to being identified with ADHD symptoms, accessing services (Bussing et al., 2003; Meyer et al., 2020; Ohan & Visser, 2009), being diagnosed with ADHD, and receiving adequate treatment (Bussing et al., 1998). The delay caused by this barrier for females to be diagnosed with ADHD could explain the levelling out of sex differences in the prevalence of ADHD in adulthood.

Sex differences in how ADHD symptoms are reported also relate to the clinical understanding and prevalence of an ADHD diagnosis. To avoid over‐ and underdiagnosis and ‐treatment, it is important to minimize referral biases related to sex and to offer appropriate treatment and follow‐up irrespective of sociodemographic factors. Mapping the distribution of ADHD symptoms across sex, age, and study populations in individuals with and without ADHD could serve as an important reference to explore possible systematic biases affecting diagnostic practice (Babinski, 2024).

Most previous reports in this field have included few and selected participants, precluding the generalizability of findings (Attoe & Climie, 2023; Degtiar & Rose, 2023). One such clinical cohort study showed that females with a diagnosis of ADHD reported significantly higher total ADHD symptom scores than males with ADHD. This sex difference was however reversed among the individuals without an ADHD diagnosis, where males reported higher ADHD scores than females (Vildalen et al., 2019). There is also a lack of studies examining how sex differences in ADHD symptom patterns vary between individuals with and without an ADHD diagnosis across the lifespan, as most studies only include participants within a narrow age span, and few studies include both clinical and population‐based samples (Kessler et al., 2006; Oerbeck et al., 2019). While a recent review of the Adult ADHD Self‐Report Scale screener (ASRS v1.1, part A) demonstrated good reliability and validity across different languages, countries, and genders/sex, the study did not differentiate between individuals with and without a clinical diagnosis of ADHD and possible sex differences in this regard (Kessler et al., 2005; Lewczuk et al., 2024). Studies including participants of both sexes, including the full range of ADHD symptoms, across age groups, in large and representative clinical and population‐based cohorts are thus called for.

Here we explored sex differences in self‐reported ADHD symptoms in adolescents and adults with and without ADHD in two Norwegian cohorts from the general population and in a clinical cohort.

## METHODS

### Study populations

This study is based on data from three non‐overlapping Norwegian cohorts; two population‐based cohorts, and one clinical cohort. The recruitment procedures for all three cohorts have been described in detail in previous publications and are briefly summarized below.

### The Norwegian Mother, Father, and Child Cohort Study (MoBa) (Magnus et al., 2016)

The Norwegian Mother, Father, and Child Cohort Study (MoBa) is a population‐based pregnancy cohort study conducted by the Norwegian Institute of Public Health. Participants were recruited from all over‐Norway from 1999 to 2008. Women consented to participation in 41% of the invited pregnancies. The cohort now includes 114,500 children, 95,200 mothers and 75,200 fathers (Magnus et al., 2016). The participants included in the present study are pregnant women and most of their partners are also included. The partners answered questions about ADHD symptoms and whether they had been diagnosed with ADHD or not at the time of inclusion in the study at the 18^th^ week of pregnancy (1999–2008). Mothers reported their own ADHD symptoms when the child was three years of age (2003–2012), and if they ever had been diagnosed with ADHD when the child was 8 years of age (2008–2018). The different timepoints for reporting ADHD symptoms and for the ADHD diagnosis were not by intentional design but related to priorities within the MoBa research group. For this study, we obtained information about ADHD symptoms from a total of 90,816 mothers and fathers (62% were mothers). The current study is based on version 12 of the quality‐assured data files released for research in January 2019.

### Youth@hordaland survey (y@h) (Skogen et al., 2019)

All adolescents born between 1993 and 1995, living in the county of Hordaland, Western Norway, during the spring 2012, were invited to participate while attending their first/second/third year in high school (11^th^/12^th^/13^th^ grade, age 16–19 years old). In the y@h‐survey, 19,489 adolescents were invited, and 53% adolescents (53% females) participated (https://www.norceresearch.no/en/projects/the‐bergen‐child‐study). The survey included questions on ADHD symptoms and whether participants had an ADHD diagnosis or not (Lundervold et al., 2016).

### The ADHD in Norwegian Adults project (ANA) (Halmoy et al., 2009)

The ADHD in Norwegian Adults (ANA) project, launched in 2004, is a study at the University of Bergen including adults who have been clinically diagnosed with ADHD at outpatient clinics or hospitals across Norway (*N* = 776, 50% females), and a population‐derived control group (*N* = 901, 60% females). The control sample in ANA (ANA controls) was mainly randomly selected from the Medical Birth Registry of Norway (MBRN) (Irgens, 2000) (79% of control sample), and partly from friends and family members of ADHD patients and from voluntarily recruited students at the University of Bergen. One of the controls reported having been diagnosed with ADHD and was excluded from the analyses, otherwise there were no exclusion criteria. All participants were included in the period of 2004–2014 and reported on ADHD symptoms when included, and for cases, close to the time of their clinical ADHD diagnosis. All ADHD diagnoses among cases were confirmed in adulthood. Birth year range for ANA were 1935–1995. Among cases mean birth year was 1972 for males, and 1973 for females, while for ANA controls, the mean birth year was 1978 for males, and 1977 for females. The participation rate for ANA cases first included was approximately 20% (338 included of 1700 invited from national registry) and additionally 457 patients were recruited directly from clinicians in the following years (Halmoy et al., 2010; Vildalen et al., 2019).

The studies were all approved by The Norwegian Regional Committees for Medical Research Ethics (2015/2055), (2013/543) and (2011/811). The STROBE criteria were followed (von Elm et al., 2007).

### ADHD diagnosis and ADHD symptom scores

For the MoBa and the y@h cohort, the ADHD diagnosis was defined from self‐reported previous or current clinical ADHD diagnosis (yes/no), here referred to as ADHD. All cases in ANA had been diagnosed with ADHD with DSM‐IV criteria by psychiatrists or clinical psychologists (Haavik et al., 2010). The 18‐item Adult ADHD Self‐Report Scale (ASRSv1.1, here called ASRS‐18) was used to assess ADHD symptoms in participants from both the y@h and ANA cohorts, while the ASRS screener version (ASRSv1.1 part A, here called ASRS‐6) (Kessler et al., 2005, 2007) was utilized in the MoBa study.

### The Adult ADHD self‐report rating scale screener 6 items (ASRS‐6)

A 6‐item screen version (ASRS‐6) has been developed based on the most frequent and predictive items of the ASRS‐18 questionnaire (Adler et al., 2006; World Health Organization, 1992). The ASRS‐6 has been used in community epidemiological surveys and in clinical outreach and case‐finding initiatives (Kessler et al., 2007), demonstrating good reliability and validity across different languages, countries, and genders (Adler et al., 2006, 2012; Hines et al., 2012; Kessler et al., 2005; Lewczuk et al., 2024). The six questions are measured on a 5‐point Likert scale (0 = never/seldom and 4 = very often), with a range in scores from 0 to 24. An ASRS‐6 score of ≥14 is commonly used as a proxy for ADHD diagnosis in studies and we used this cut‐off in the three different cohorts, stratified by the presence of a reported diagnosis of ADHD and sex (Kessler et al., 2007).

### Statistical analyses

We used linear regression models to analyse sex differences in continuous levels of ASRS‐6 scores between adolescents and adults with and without a reported ADHD diagnosis in the three cohorts (stratifying by sex). Interaction between sex and ADHD was included in the model. Linear regression models were also used to analyse differences in ASRS‐6 scores between males and females (females as reference). We adjusted for age and in the MoBa cohort we used the cluster (variable) function in Stata 16.1 to account for correlations between parents. Overlap in age was due to overlapping age groups in the cohorts. We further calculated the proportion of individuals with ASRS‐6 score of ≥14 among males and females with and without and ADHD diagnosis. As a sensitivity analysis, we also analysed differences in continuous levels of ASRS‐18 scores between adolescents and adults with and without ADHD for males and females separately, available for the ANA and y@h cohorts. Unstandardized regression coefficients (b) with 95% confidence intervals (95% CI), adjusted for age, were reported. Analyses using STATA version 16.1 (StataCorp, 2015. StataCorp LP) were conducted between March 2023 and December 2023.

## RESULTS

### Descriptive information from the cohorts

From the MoBa cohort, we obtained information about ADHD symptoms from a total of 56,426 (62%) mothers, and 34,390 (38%) fathers. The mean age of reporting ADHD symptoms for mothers (when child was 3 years) was 33.4 (SD (standard deviation) 4.4, range 20–48 years), and for fathers (at 18 weeks pregnancy) 32.9 (SD 5.3, range 18–59 years). In MoBa, both females and males with ADHD were significantly younger at childbirth than those without ADHD, and thus younger when answering the questionnaires, with a difference in mean age of 0.8 (SD 0.12) years for females and 4.0 (SD 0.45) years for males. In the y@h cohort, we obtained data from 10,257 individuals (5401; 53% females). Mean age for males and females in the y@h cohort were similar (17.4/17.5 years), with an age range of 16–19 years, both for those with and without ADHD (Table 1). In the ANA cohort, the ANA controls were younger than the ANA cases (Table 1). The age ranges for ANA controls were 18–74 years for females/18–58 years for males, while for ANA cases age ranged from 17 to 71 years for females/18–67 years for males.

**TABLE 1:** Descriptive information of adolescents and adults in the included cohorts.

	MoBa	y@h	ANA
Number of participants	N (%)		
Total	90,816	10,257	1677
Females	56,426 (62.1)	5401 (52.7)	930 (55.4)
Males	34,390 (37.9)	4856 (47.3)	747 (44.5)
ADHD diagnosis	328 (0.4)	197 (1.9)	776 (46.4)
Females	182 (0.3)	88 (1.6)	390 (50.1)
Males	146 (0.4)	109 (2.2)	386 (49.6)
Mean age without ADHD	Years (SD)		
Females	33.4 (4.4)	17.5 (0.84)	29.0 (7.8)
Males	32.9 (5.3)	17.4 (0.84)	29.9 (7.3)
Mean age with ADHD	Years (SD)		
Females	29.0 (5.2)	17.5 (0.84)	34.8 (10.1)
Males	28.9 (5.4)	17.4 (0.84)	33.6 (10.5)

Abbreviations: ANA, ADHD in Norwegian Adult study; MoBa, the Norwegian Mother, Father and Child Study; SD, standard deviation; y@h, youth@hordaland study.

### Sex difference in mean ASRS‐6 scores in adolescents and adults with and without reported ADHD

**FIGURE 1:**
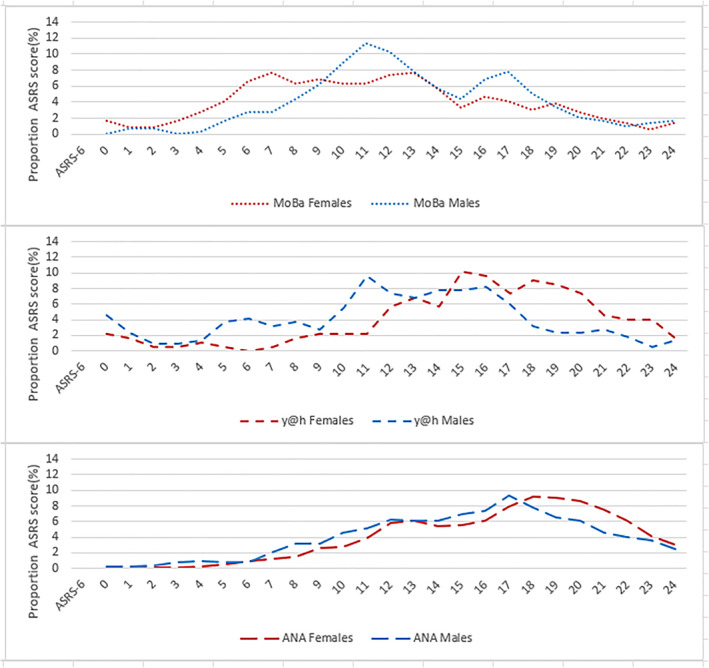
Distribution of ASRS‐6 score by sex for adolescents and adults with ADHD in all cohorts. ANA, ADHD in Norwegian Adult study; MoBa, the Norwegian Mother, Father and Child Study; y@h, youth@hordaland study.

**FIGURE 2:**
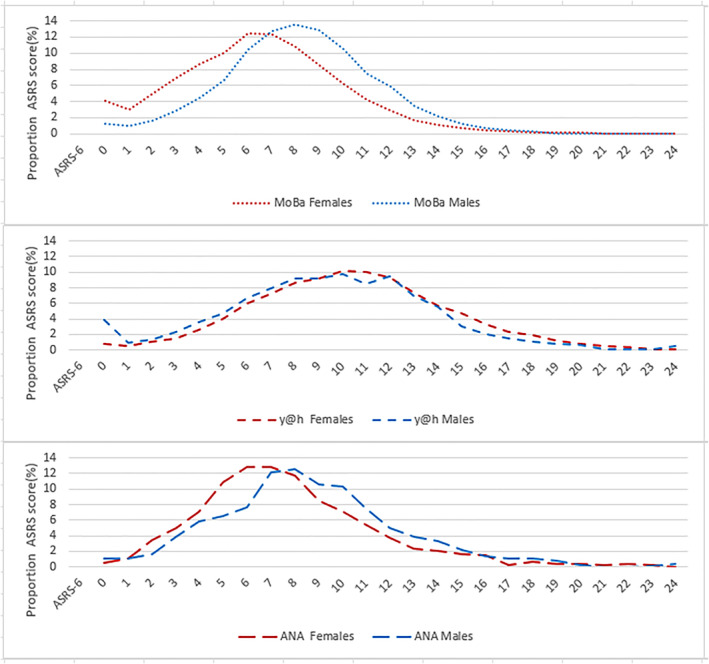
Distribution of ASRS‐6 score by sex for adolescents and adults without ADHD in all cohorts. ANA, ADHD in Norwegian Adult study; MoBa, the Norwegian Mother, Father and Child Study; y@h, youth@hordaland study.

**FIGURE 3:**
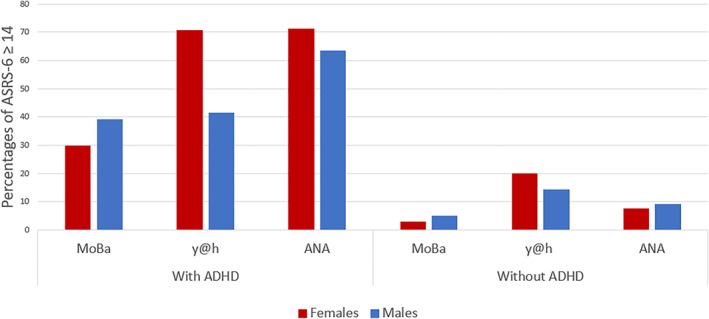
Percentage of adolescents and adults with ASRS‐6 score equal to or above 14 in all cohorts. ANA, ADHD in Norwegian Adult study; MoBa, the Norwegian Mother, Father and Child Study; y@h, youth@hordaland study.

The distribution of ASRS‐6 scores in adults and adolescents with and without ADHD is shown by cohort in Figures 1 and 2, respectively. The younger males and females without ADHD from the y@h cohort reported higher ASRS‐6 symptom scores compared to persons without ADHD in the other two cohorts, Figure 1. Among participants without ADHD, approximately 20% of females and 14% of males in the y@h cohort reported a score equal to or above 14, compared to between 3% and 8% of females and 5%–9% of males in the two other cohorts (Table 2/Figure 3).

**TABLE 2:** Adolescents and adults without and with ADHD and the proportion with ASRS‐6 equal to or above 14 in all cohorts.

	MoBa N (%)	y@h N (%)	ANA N (%)
Without ADHD	3317 (3.7)	1729 (16.9)	73 (8.1)
Females	1624 (2.9)	1053 (19.8)	41 (7.6)
Males	1693 (4.9)	676 (14.2)	32 (8.9)
With ADHD	111 (33.8)	107 (54.3)	523 (67.1)
Females	54 (29.7)	62 (70.5)	278 (71.3)
Males	56 (38.9)	45 (41.3)	245 (63.5)

Abbreviations: ANA, ADHD in Norwegian Adult study; MoBa, the Norwegian Mother, Father and Child Study; y@h, youth@hordaland study.

In MoBa, both among those without and with ADHD, males had significantly higher mean ASRS‐6 scores than females: without ADHD (*b* = 1.7 (CI 1.7; 1.8, *p* < *0.001*)); with ADHD (*b* = 1.5 (CI 0.4; 2.6, *p* = *0.007*)).

In the y@h cohort, males had a lower mean ASRS‐6 score than females both among those without and with ADHD: without ADHD (*b* = ‐ 1.2 (CI ‐1.3; −1.0, *p* < *0.001*)), with ADHD (*b* = −3.0 (CI ‐4.6; −1.5, *p* < *0.001*)).

In the ANA control cohort, we found no significant difference in mean ASRS‐6 symptom score between males and females without ADHD (*b* = 0.5 (CI ‐0.02; 1.0, *p* = *0.062*)), but a significantly lower mean score in males with ADHD than in females with ADHD (*b* = −1.3 (CI ‐2.0; −0.7, *p* < *0.001*)) (Table 3).

**TABLE 3:** Calculated differences of mean ASRS‐6 scores by sex for adolescents and adults with/without ADHD and within each sex with/without ADHD.

ASRS‐6	With ADHD	Without ADHD	∆ with/without ADHD
	Mean (SD)	Mean (SD)	Adjusted for age
MoBa			
Females	11.2 (5.4)	6.6 (3.4)	*b* = 4.6 (3.8; 5.4) *p* < *0.001*
Males	12.7 (4.6)	8.2 (3.2)	*b* = 4.2 (3.5; 5.0) *p* < *0.001*
∆ females/males adj for age	*b* = 1.5 (0.4; 2.6) *p* = *0.007*	*b* = 1.7 (1.7; 1.8) *p* < *0.001*	
y@h			
Females	15.2 (5.0)	10.1 (4.4)	*b* = 5.1 (4.0; 6.2) *p* < *0.001*
Males	12.0 (5.5)	9.0 (4.3)	*b* = 3.2 (2.1; 4.2) *p* < *0.001*
∆ females/males adj for age	*b* = −3.0 (−4.6; −1.5) *p* < *0.001*	*b* = −1.2 (−1.3; −1.0) *p* < *0.001*	
ANA			
Females	16.2 (4.5)	7.7 (3.7)	*b* = 8.6 (8.1; 9.2) *p* < *0.001*
Males	14.9 (4.9)	8.2 (3.9)	*b* = 6.5 (5.9; 7.2) *p* < *0.001*
∆ females/males adj for age	*b* = −1.3 (−2.0; −0.7) *p* < *0.001*	*b* = 0.5 (−0.02; 1.0) *p* < *0.001*	

Abbreviations: ANA, ADHD in Norwegian Adult study; MoBa, the Norwegian Mother, Father and Child Study; y@h, youth@hordaland study. ∆ = difference; b – unstandardized regression coefficient for test of difference with 95% CI and *p*‐values.

### Difference in mean ASRS‐6 scores in adolescents and adults with and without ADHD, by sex and study cohort

**FIGURE 4:**
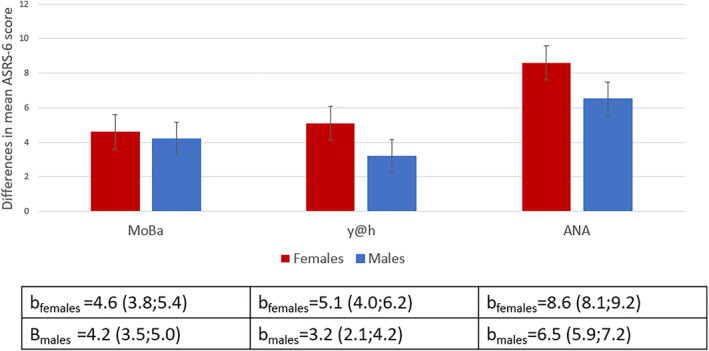
Differences in mean ASRS‐6 score between adolescents and adults with and without ADHD, by sex, adjusted for age. ANA, ADHD in Norwegian Adult study; MoBa, the Norwegian Mother, Father and Child Study; y@h, youth@hordaland study.

In the y@h and ANA cohorts, the differences in mean ASRS‐6 scores between females with versus without ADHD were larger than the corresponding differences in males: (y@h: ∆females: *b* = 5.1 (CI 4.0; 6.2); ∆males: *b* = 3.2 (CI 2.1; 4.2); ANA: ∆females: *b* = 8.6 (CI 8.1; 9.2); ∆males: *b* = 6.5 (CI 5.9; 7.2)). In the MoBa cohort, this difference was less pronounced with 4.6 in females and 4.2 in males (∆females: *b* = 4.6 (CI 3.8; 5.4); ∆males: *b* = 4.2 (CI 3.5; 5.0)) (Table 3 and Figure 4).

Testing for interaction by sex showed that the difference in results between males and females in the ANA cohort was statistically significant (*p* for interaction <0.001), while this was not so in the MoBa cohort (*p* for interaction: 0.519). Formal interaction testing was not possible in the y@h cohort, as data were no longer available, however, confidence intervals only slightly overlapped for males and females.

The patterns in differences in mean ASRS‐18 scores in those with and without ADHD by sex for the y@h and ANA cohorts were found to be similar to the ASRS‐6 (y@h: ∆females: *b* = 14.1 (CI 11.3; 17.0); ∆males: *b* = 9.4 (CI 6.9; 11.9); ANA ∆females: *b* = 23.7 (CI 22.1; 25.2); ∆males: *b* = 18.9 (CI 17.2; 20.6)). The results are shown in Supporting Information, Table S1.

## DISCUSSION

### Main findings

As expected, both males and females diagnosed with ADHD reported much higher ADHD symptom scores than participants without ADHD. However, the differences in symptom scores between individuals with and without ADHD were larger for females than for males. These findings were consistent and statistically significant across two of the three cohorts examined: A large population‐based cohort of adolescents, and a clinical cohort of adults with and without an ADHD diagnosis. This pattern of sex difference was similar, but less striking in the third sample, the population‐based MoBa cohort. Further, the sex‐based differences in reported symptom severity between females with and without ADHD was largest in the clinical sample.

These findings could suggest that females may require a higher symptom load to be diagnosed with ADHD. Further, our results suggest that the threshold for obtaining an ADHD diagnosis, in terms of self‐reported ADHD symptoms, may be higher in females than in males, as the difference was most pronounced in the clinical cohort.

There are several possible interpretations for our findings. Since we have no information about the clinical decisions used to define the individual ADHD cases, we don't know whether males and females are assessed differently across the remaining diagnostic criteria for ADHD (e.g. age of onset and impairment). However, if differences in assessment and diagnostic procedure is the case, it could indicate a systematic barrier towards diagnosing ADHD in females and that the diagnostic threshold in clinical practice is higher for females than for males, as previously suggested (Bussing et al., 2003; Meyer et al., 2020; Ohan & Visser, 2009). Our finding of a larger difference in symptom severity between individuals with and without ADHD in females in the clinical cohort both as compared to males in the same cohort and to the other cohorts may support this interpretation. Such differences were also reported in a study of Vildalen et al. where they separately analysed symptoms of inattention and hyperactivity/impulsivity using the full 18 item ADHD symptom questionnaire (ASRS‐18) (Vildalen et al., 2019).

Sex differences in diagnosis of ADHD may be explained by a larger proportion of females with high ADHD symptom scores who do not meet other diagnostic criteria such as age of onset and impairment. If we assume that diagnostic procedures are similar for males and females, this may indicate that females could be less impaired by their ADHD symptoms due to compensatory strategies, have their ADHD symptoms attributed to other mental disorders with overlapping symptom profiles, or they may receive an ADHD diagnosis later in life (Halmoy et al., 2009; Skoglund et al., 2023). This might be a contributing factor in the y@h‐cohort, where 19.8% of the females, but only 14.2% of the males without a diagnosis scored above the ASRS threshold (Table 2).

Previous studies of children, youths, and adults have suggested that females face systematic barriers to obtaining an ADHD diagnosis (Attoe & Climie, 2023; Bussing et al., 2003; Meyer et al., 2020; Ohan & Visser, 2009; Young et al., 2020). Such a barrier could cause females who qualify for an ADHD diagnosis to suffer with larger impairment and to require higher levels of ADHD symptoms before the problems are perceived by themselves and others as being related to ADHD. It has also been suggested that females with ADHD are particularly susceptible to be missed during the ADHD diagnostic process and less likely to be prescribed medication if they don't show prominent externalising problems (Attoe & Climie, 2023; Mowlem et al., 2019; Mowlem et al., 2019; F. D. Mowlem et al., 2019) which has been traditionally associated with an ADHD diagnosis in males. Females thus need to report more symptoms than males to receive a clinical diagnosis of ADHD (Hinshaw et al., 2022; Mowlem et al., 2019; Mowlem et al., 2019; Williamson & Johnston, 2015), aligning with the findings in the present study.

There may also be differences in reporting styles in females and males. Females are in general more prone to report symptoms than males (Ladwig et al., 2000). However, the fact that females without ADHD in our study report less symptoms than males without ADHD does not support this as an explanation for sex differences in ADHD symptoms. It has been observed that females with ADHD report symptoms as being more severe at the same impairment level as males (Biederman et al., 2004; Williamson & Johnston, 2015). Females with high levels of ADHD symptoms may report higher scores than their male counterparts because they deviate more from their sex‐relative population mean, as observed in this study. This is supported by a study of children, where females referred for and diagnosed with ADHD exhibited symptoms that significantly deviated more from their expected behaviours compared to males (F. D. Mowlem et al., 2019b). Further, this is also in line with studies where females with ADHD reported feeling different from females without ADHD (Attoe & Climie, 2023). This difference in self‐evaluation/self‐reporting styles may stem from a biological/genetic difference between males and females and by stigma and socio‐cultural factors/expectations, and is shown across a range of mental health problems and subjective wellbeing (Yoon et al., 2023). For example, clinicians may fail to see or acknowledge depressive symptoms in a restless male and impulse control symptoms in a depressed female (Vildalen et al., 2019).

The less pronounced sex‐difference in MoBa could be related to selection bias and differential attrition. First, mothers had to consent to participate and then invite the father of the child to participate. Further, the ADHD symptoms of the mother were only recorded at the 3‐year questionnaire whereas the fathers reported ADHD symptoms at inclusion (gestational week 18). To remain in such a study over time is challenging, in particular for people with ADHD. The relatively lower mean ASRS scores in MoBa females both for those with and without ADHD compared to the other two cohorts could indicate a selection bias associated with higher loss to follow‐up in females with more severe ADHD symptoms. In contrast, the males in MoBa had similar mean ASRS score as males in the other two cohorts. As these fathers reported their symptoms at the time of inclusion, they were probably subject to less attrition. Further, while the MoBa cohort initially included 42% of those invited, only 22% remained when the children were 8 years of age. The study may have selection bias because participants with more severe ADHD symptoms were less likely to join (Biele et al., 2019). This could explain why the study found fewer reported ADHD symptoms among adults in MoBa than expected, suggesting the participant group may not be representative for the general population of parents in Norway. Thus, selection bias may partly explain the non‐significant difference between mothers and fathers in MoBa with and without ADHD.

### Strengths and limitations

Our study has several strengths, including large samples with information about ADHD symptoms, including both sexes, spanning age groups between 16 and 74 years. Furthermore, the cohorts represent parents and non‐parents, self‐reported and clinically validated ADHD, and a large sample of adolescents and adults without ADHD recruited from both the general and clinical adult Norwegian population, with data from >100,000 individuals.

However, there are limitations to consider. Selection bias may be present in all three studies. We know from previous studies that the socio‐economic status of MoBa participants at the time of recruitment was higher than that of the average Norwegian population (Biele et al., 2019; Magnus et al., 2016). Moreover, the ANA cases are on the opposite spectrum, with lower socio‐economic status and a higher genetic pre‐disposition for ADHD. For the y@h cohort, about 50% of the invited adolescents participated which could also affect the generalizability of these findings. In a previous study from this cohort, non‐participants were found to have more psychological problems than participants (Hysing et al., 2016; Stormark et al., 2008). It is therefore likely that the prevalence of mental health problems or, in this case, ADHD symptoms/diagnosis, could be underestimated in the present study.

Unfortunately, our data was not linked to registries with information on ADHD diagnosis or medication which could have strengthened the validity of the ADHD diagnosis. Furthermore, since the ADHD diagnosis was defined as ADHD “ever” in the MoBa and y@h cohorts, the ASRS‐6 score does not necessarily reflect the ADHD symptoms at the time of diagnoses. This could be especially true for the MoBa cohort, where participants may have been treated or “grown out of” an ADHD diagnosis as they have answered the question “have or ever had ADHD”. Since they would be defined as having ADHD in our study, this could partly explain that the difference in ASRS‐6 scores between those with and without ADHD was slightly smaller in MoBa than in the other cohorts, at least for females. We do not have any reason to assume that the diagnostic threshold differed systematically across the cohorts. Females may thus have been relatively underdiagnosed in all three cohorts.

A strength of our study is that the cohorts together include a wide age‐range spanning from adolescence to older adulthood. The higher symptom‐score found in the youngest cohort is in line with previous findings of a general decline of ADHD symptoms with age (Wootton et al., 2022). Future studies including also children might be needed to assess this further.

The ASRS data used in this study were collected during 1999–2011. After the COVID‐19 pandemic, the incidence and prevalence of ADHD and some other psychiatric disorders have increased significantly in many countries, including Norway (Gimbach et al., 2024). This increase has been particularly strong among young females, where the incidence of ADHD for example, in Norway and Finland in 2022 surpassed the corresponding incidence in males (Auro et al., 2024; Hartz et al., 2024). It is unclear whether this development reflects a real increase or that sex‐related barriers towards this diagnosis are becoming less apparent. Some caution is thus warranted before generalizing our results to the current diagnostic situation, and future studies are needed to clarify whether there has been a real change in clinical practice or not in these latest years.

In our analyses we analysed sex differences as a dichotomous variable since this was the available variable in our data, and we have cited different studies reporting (biological) sex and gender manifestations of sex. However, human sex differences and the sex of the human brain may also be conceptualized on a continuum, including the sex‐specific presentation of psychopathology and its relationship with genetic variants (Phillips et al., 2019).

## CONCLUSIONS

This study indicated that individuals with ADHD report more ADHD symptoms than individuals without ADHD and that these differences in symptom scores were larger for females than for males. We identified sex differences in self‐reported ADHD symptoms in both population‐based and clinical cohorts across age groups and ADHD diagnosis. The relatively higher severity level of ADHD symptoms in females compared to males may infer that there are barriers for females related to referral, assessment, and diagnosis of ADHD. From a clinical perspective, it is important to be aware of different presentations between males and females to ensure appropriate level of treatment and support.

## AUTHOR CONTRIBUTIONS

**Berit S. Solberg:** Conceptualization; data curation; formal analysis; funding acquisition; methodology; project administration; visualization; writing—original draft; writing—review and editing. **Maj‐Britt Posserud:** Data curation; formal analysis; writing—original draft; writing—review and editing. **André Høberg:** Data curation; methodology; writing—original draft; writing—review and editing. **Johanne T. Instanes:** Formal analysis; methodology; writing—original draft; writing—review and editing. **Liv G. Kvalvik:** Conceptualization; methodology; writing—original draft; writing—review and editing. **Amalie Austgulen:** Methodology; visualization; writing—original draft; writing—review and editing. **Ammal Khan:** Methodology; writing—original draft; writing—review and editing. **Astri J. Lundervold:** Methodology; writing—original draft; writing—review and editing. **Anne Halmøy:** Methodology; writing—original draft; writing—review and editing. **Rolf Gjestad:** Formal analysis; methodology; writing—original draft; writing—review and editing. **Kari Klungsøyr:** Formal analysis; methodology; writing—original draft; writing—review and editing. **Jan Haavik:** Conceptualization; funding acquisition; project administration; supervision; visualization; writing—original draft; writing—review and editing.

## CONFLICT OF INTEREST STATEMENT

During the past 3 years, J.H. has received lecture honoraria as part of continuing medical education programs sponsored by Medice all outside the submitted work. B.S.S. has received honoraria as part of medical education programs sponsored by Takeda and Medice; all outside the submitted work. The other authors declare no conflict of interest.

## ETHICAL CONSIDERATIONS

The studies were all approved by The Norwegian Regional Committees for Medical Research Ethics (2015/2055), (2013/543) and (2011/811). The STROBE criteria were followed (von Elm et al., 2007).

## Supporting information

Supporting Information S1

## Data Availability

The data that support the findings of this study are available from MoBa, y@h and ANA. Restrictions apply to the availability of these data, which were used under license for this study. Data from the Norwegian Mother, Father and Child Cohort Study and the Medical Birth Registry of Norway used in this study are managed by the national health register holders in Norway (Norwegian Institute of public health) and can be made available to researchers, provided approval from the Regional Committees for Medical and Health Research Ethics (REC), compliance with the EU General Data Protection Regulation (GDPR) and approval from the data owners. The consent given by the participants does not open for storage of data on an individual level in repositories or journals. Researchers who want access to data sets for replication should apply through helsedata. no. Access to data sets requires approval from The Regional Committee for Medical and Health Research Ethics in Norway and an agreement with MoBa.
