# Supplementary material for: Sex differences in self‐reported attention‐deficit/hyperactivity disorder symptoms in clinical and population‐based cohorts
Source: JCPP Adv. 2025 Mar 26;5(4):e70012. doi: 10.1002/jcv2.70012 (PMC12698277; doi:10.1002/jcv2.70012)
Supplement: Supplementary file 1 — Supporting Information S1 [file JCV2-5-e70012-s001.docx]

**Supporting Information**

**Table S1. Calculated differences of mean ASRS-18 scores by sex in adolescents and adults with/without ADHD and within each sex with/without ADHD**

| **ASRS-6** | **With ADHD**  **Mean (SD)** | **Without ADHD**  **Mean (SD)** | **∆ with/without ADHD**  **Adjusted for age** |
| --- | --- | --- | --- |
| **y@h** |  |  |  |
| **Females** | 41.8 (13.4) | 27.7 (11.1) | b=14.1 (11.3;17.2) *p<0.001* |
| **Males** | 32.8 (13.2) | 23.6 (12.0) | b=9.4 (6.9;11.9) *p<0.001* |
| **∆ females/males adj for age** | b= -8.4 (-12.2; -4.6) *p<0.001* | b= -4.0 (-4.4; -3.5) *p<0.001* |  |
| **ANA** |  |  |  |
| **Females** | 46.1 (12.3) | 22.6 (9.8) | b=23.7 (22.1;25.2) *p<0.001* |
| **Males** | 42.4 (13.1) | 23.2 (9.7) | b=18.9 (17.2;20.6) *p<0.001* |
| **∆ females/males adj for age** | b= -3.6 (-5.4; -1.8) *p<0.001* | b=0.6 (-0.7;1.9) *p=0.367* |  |

MoBa – the Norwegian Mother, Father and Child Study; y@h – youth@hordaland study; ANA – ADHD in Norwegian Adult study; ∆= difference; b – unstandardized regression coefficient for test of difference with 95%CI and p-values
